# Win, Draw, or Lose? Global Positioning System-Based Variables’ Effect on the Match Outcome: A Full-Season Study on an Iranian Professional Soccer Team

**DOI:** 10.3390/s21175695

**Published:** 2021-08-24

**Authors:** Hadi Nobari, Norbert Keshish Banoocy, Rafael Oliveira, Jorge Pérez-Gómez

**Affiliations:** 1 Department of Physical Education and Sports, University of Granada, 18010 Granada, Spain; 2 HEME Research Group, Faculty of Sport Sciences, University of Extremadura, 10003 Cáceres, Spain; 3 Department of Exercise Physiology, Faculty of Sport Sciences, University of Isfahan, Isfahan 81746-7344, Iran; 4 Sports Scientist, Sepahan Football Club, Isfahan 81887-78473, Iran; 5 School of Medical and Health Sciences, Edith Cowan University, Perth 6027, Australia; norbert.keshish@gmail.com; 6 Physical Performance Department, Helsingin Jalkapalloklubi (Football Club of Helsinki), 00250 Helsinki, Finland; 7 Institute of Santarém, Sports Science School of Rio Maior–Polytechnic, 2140-413 Rio Maior, Portugal; rafaeloliveira@esdrm.ipsantarem.pt; 8 Research Center in Sport Sciences, Health Sciences and Human Development, Quinta de Prados, Edifício Ciências de Desporto, 5001-801 Vila Real, Portugal; 9 Life Quality Research Centre, 2140-413 Rio Maior, Portugal

**Keywords:** acceleration, deceleration, performance, GPS, fatigue, situational variables, WIMU, win

## Abstract

The aim of the study was to determine the between-match and between-halves match variability of various Global Positioning System (GPS) variables and metabolic power average (MPA) in competitions, based on the match results obtained by professional soccer players over a full season. Observations on individual match performance measures were undertaken on thirteen outfield players competing in the Iranian Premier League. The measures selected for analysis included total duration, accelerations in zones (AccZ1, 2, and 3), decelerations in zones (DecZ1, 2, and 3), and MPA collected by the Wearable Inertial Measurement Unit (WIMU). The GPS manufacturer set the thresholds for the variables analyzed as follows: AccZ1 (<2 m·s^−2^); AccZ2 (2 to 4 m·s^−2^); AccZ3 (>4 m·s^−2^); DecZ1 (<−2 m·s^−2^); DecZ2 (−2 to −4 m·s^−2^); DecZ3 (>−4 m·s^−2^). The results revealed significant differences between wins and draws for the duration of the match and draws compared to wins for the first- half duration (*p* ≤ 0.05; ES = 0.36 [−0.43, 1.12]), (*p* ≤ 0.05; ES = −7.0 [−8.78, −4.78], respectively. There were significant differences on AccZ1 during the first-half between draws and defeats (*p* ≤ 0.05; ES = −0.43 [−1.32, 0.46]), for AccZ3 in the second-half between draws and defeats (*p* ≤ 0.05; ES = 1.37 [0.48, 2.25]). In addition, there were significant differences between wins and draws (*p* ≤ 0.05; ES = 0.22 [−0.62, 1.10]), and wins and defeats for MPA in the first- half (*p* ≤ 0.05; ES = 0.34 [−0.65, 1.22]). MPA showed further differences between draws and defeats in the second- half (*p* ≤ 0.05; ES = 0.57 [−0.22, 1.35]). Descriptive analysis revealed differences between the first and second half for wins in AccZ2 (*p* = 0.005), DecZ2 (*p* = 0.029), and MPA (*p* = 0.048). In addition, draws showed significant differences between the first and second half in duration, AccZ1, AccZ2, and DecZ2 (*p* = 0.008), (*p* = 0.017), (*p* = 0.040), and (*p* = 0.037) respectively. Defeats showed differences between the first and second half in AccZ1, AccZ3, and MPA (*p* = 0.001), (*p* = 0.018), and (*p* = 0.003) respectively. In summary, the study reveals large variations between the match duration, accelerometer variables, and MPA both within and between matches. Regardless of the match outcome, the first half seems to produce greater outputs. The results should be considered when performing a half-time re-warm-up, as this may be an additional factor influencing the drop in the intensity markers in the second half in conjunction with factors such as fatigue, pacing strategies, and other contextual variables that may influence the results.

## 1. Introduction

The influx of micro-technology, such as Wearable Inertial Measurement Units (WIMU) and Electronic Performance Tracking Systems (EPTS) within the practice setting, has increased the ways practitioners can monitor and analyze training load through different measures such as total distance covered, distance in different speed zones, accelerations, decelerations, heart rate, etc. [1]. Recent growth for various match analysis techniques has been highlighted with a systematic review conducted by Sarmento et al., (2018) [2]. The increased understanding of the technical and tactical behavior of the teams and players across varying levels can result in addressing the needs of training and improving practice design [2]. This improved technical and tactical understanding needs to be in conjunction with the physical requirements underpinning the collective behavior of the team during matches and practices. 

In a study by Barrera et al. [3] on Portuguese soccer players, the match location and the results of the match impacted the physical demands of each playing position, specifically with a decrease in the second half of the matches. A recent international survey that evaluated the current practices and perceptions in high-level soccer found that accelerations, at various thresholds, and estimated metabolic power average (MPA) were in the top five training load variables used [1]. High-intensity accelerations and decelerations make up a large portion of the workload profile within the competitive match play, and they impose a unique biomechanical and physiological load on players [4]. In a three-season study conducted on an elite Norwegian professional team, it was shown that accelerations make up 7–10%, and decelerations 5–7% of the total player load across various playing positions in the match [5]. The overall metabolic cost of accelerations seems to be higher, while a higher mechanical load is associated with decelerations likely due to high impact peaks and loading rates [5,6,7]. Therefore, decelerations may increase the likelihood of soft tissue injuries due to high forces not attenuated in an efficient manner [8]. 

In addition to being indicative of muscle damage post-match, the reductions in neuromuscular performance capacity are associated with high-intensity accelerations and decelerations frequencies during the matches [9,10,11,12]. Overall, there is a higher number of high-intensity decelerations compared to high-intensity accelerations, along with a reduction from the first to the second half in the competitive match for both variables [4]. As a result, there is a potential link to neuromuscular fatigue and a greater risk of injury. The reduction of high and very high-intensity accelerations and decelerations, between the first and second half in the competitive match, is important to consider for the evaluation of training sessions, and the potential role in the preparation of players for the competitive fixtures. 

In a recent study on female Portuguese soccer players, match running performance in the context of accelerations and decelerations (i.e., high-intensity actions) were significantly predicted by squat and countermovement jump and change of direction ability. In addition, there was a large to a very large correlation between maximal sprinting capability and high-intensity match actions [13]. To gain a better understanding of what underlying physical capacities are essential for match preparation and match-running performance, analysis of the match physical output in relation to the outcome of the match may be warranted to provide further contextualization and subsequent physical preparation. 

As mentioned previously, accelerations and decelerations are an integral part of a soccer match [4,14]. They impose a great metabolic load every time acceleration is elevated, even at low speeds, and not only during maximal intensive phases when speeds are higher [14]. In addition, the concept of MPA proposed by di Pampero et al. [15], and later by Ogsnach et al. [14] estimated the energetic cost of accelerated and decelerated running, by showing accelerated running on flat terrain is energetically equivalent to uphill/downhill running at a constant speed, where the angle of incline is equivalent to the extent of the forward acceleration [14,15,16]. In this method, an equivalent slope is provided in which the instantaneous energy cost of accelerated running and an estimate of MPA output is derived [14,17,18]. This approach showed to be a useful method of video match analysis, as a means of re-defining what qualifies as a high-intensity movement. 

Global Positioning Systems (GPS) have further allowed the estimation of energy cost, overall energy expenditure, and instantaneous MPA, from which effective oxygen consumption can also be derived [15]. In a study by Manzi et al. [19], MPA was found to be largely associated with aerobic fitness variables such as oxygen consumption and maximal aerobic speed. The results further highlight the importance of this novel concept, as a means of investigating match play and quantifying possible match-winning moments. Moreover, as recently reported, the match results and workload should be quantified for better adjustments in training sessions, in order to recover players from the last match and prepare them for the next one [20]. Furthermore, this serves as an additional metric to evaluate the fitness of players in relation to the drop in intensity from the first half to the second half, in conjunction with the contextual variables that may affect the outcome. 

The concept of MPA later adopted as a metric utilized by WIMU and often integrated within EPTS, has been an equivocal topic regarding validity and reliability, and the overall accuracy of the information regarding energy expenditure provided by the GPS [17,21,22,23,24,25,26]. As technological advancements grow with a greater capability to increase sampling frequencies, and more research validating the higher frequency GPS units [24,25,26,27], more practitioners and researchers can incorporate metrics that go beyond the one-dimensional metrics, such as distances covered and various speed zones. As new literature emerges regarding a more integrated approach in contextualizing training and match loads [28,29] and first and second halves [27], it is becoming more prevalent to gain a better understanding of the demands associated with different scenarios of matches, before layering in the technical/tactical dimension to further integrate the different dimensions of the game that can influence the match outcome. In addition, game running performance has also been linked with game performance indicators and with positional specificity [30]. The aforementioned approach can provide further context regarding the preparation practices throughout the training sessions in relation to the context of the matches. Hence, the use of micro-electro-mechanical technology (i.e., GPS, local positioning system, WIMU) to monitor and manage external loads in training and soccer matches has increased after FIFA enacted regulations by recorded WIMU and EPTS in soccer competitions [31,32]. Although studies have been conducted since then [32,33,34], more studies are needed due to variations within the different levels of competitions across leagues of different countries. A recent study done by Nobari et al. [29] highlights an example of external workload comparison in relation to the match outcome, as well as within-match analysis of the chosen variables with respect to the first and second halves. In another study conducted by Nobari et al. (2021), differences in the training load between starters and non-starters were found [33]. This further highlights the importance of identifying physical outputs of match-play as a significant variation may exist between the playing squad, which can influence the physical preparation strategies. Furthermore, Clemente et al. (2021) highlighted the influence of congested fixtures in the Portuguese league on the mechanical work of starters and non-starters in the form of high accelerations, decelerations, and high-metabolic load distance [32]. This study further supports the quantification of selected variables between the first and second half across various leagues and competitive levels, and between matches to further allow for monitoring of the squad in relation to the competitive schedule and mitigation of injury risk via implementation of recovery strategies or more aggressive squad rotation. 

Therefore, we hypothesized that depending on the outcome of matches, such as win, draw, or defeat, players may have different performances on acceleration, deceleration, and MPA. Furthermore, the values of these variables can vary from the first to the second half. With this hypothesis, the aims of this study were to: (i) compare the external workload through total duration, accelerations in zone 1 (AccZ1), accelerations in zone 2 (AccZ2), accelerations in zone 3 (AccZ3), decelerations in zone 1 (DecZ1), decelerations in zone 2 (DecZ3), decelerations in zone 3 (DecZ3), and MPA by match based on their results (i.e., win, draw or defeat); (ii) compare the same external workload measures between first and second halves in soccer players from the Iranian Premier League (IPL).

## 2. Materials and Methods

### 2.1. Experimental Approach to the Problem

**Figure 1:**
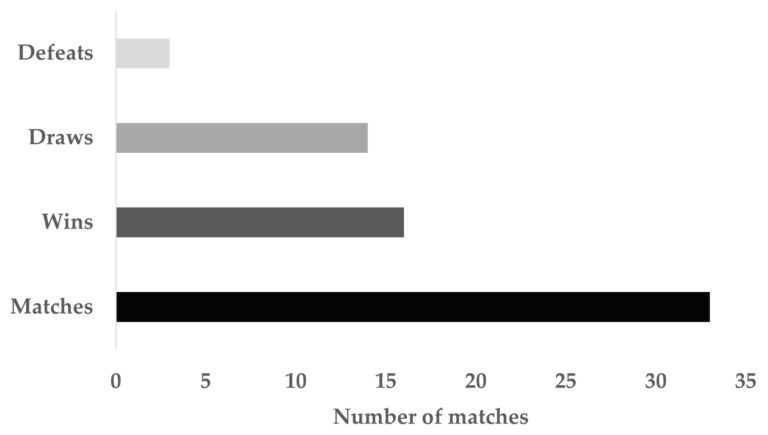
The number of matches evaluated and their division according to their results during the full season.

This study included data collected during the full season of 2018–2019. An IPL professional soccer team participated in this study. During the competitive season, the team participated in the Persian Gulf Premier League and knockout tournament. From a total of 33 matches, 16 wins, 14 draws, and 3 defeats occurred in the season (Figure 1). All matches were analyzed through a GPS (GPSPORTS systems Pty Ltd., Model: SPI High-Performance Unit (HPU); Canberra, Australia. 

### 2.2. Participants

From a total of 24 players, 13 players participated in this study. According to previous studies, to be included in the study, participants must participate in a minimum of three training sessions each week and they need to complete at least 60 min in three consecutive matches (seven players were removed based on this criterion) [35,36]. If players get injured or did not participate in training for at least two consecutive weeks (two players were removed based on this criterion), they would be excluded. In addition, due to the positional differences, the two goalkeepers were also excluded from the study. Before the start of the season, the study protocol was explained to players. Then, all player provided they written consent. The Ethics Committee of the University of Isfahan (IR.UI.REC.1399.064) approved the study that was conducted according to the ethical guidelines of the Declaration of Helsinki for studies involving humans.

#### Sample Size

Due to the small sample size, the sample power of the post hoc F-test family was calculated for α level = 0.05; effect size = 0.6; three groups, and *n* = 13 through by the G-Power [37]. It was shown that there was a 97.8% (actual power) for the analysis. In addition, another sample power calculation was made for a post hoc T-test family for α level = 0.05; effect size = 0.8; and *n* = 13 which revealed 85% of actual power.

### 2.3. Monitoring External Workload

#### 2.3.1. GPS Receiver Specifications

As mentioned before, data was collected through a 15 Hz GPSPORTS systems Pty Ltd (model SPI HPU) for professional athletes (Canberra, Australia). This GPS presented a high validity and reliability [26]. During data collection, there were no reported adverse weather conditions. 

#### 2.3.2. Data Collection by Wearable Inertial Measurement Unit

Data exported from SPI HPU GPS-based tracking systems for professional athletes, which offered 15 Hz position GPS was collected with the considerations of previous studies [35,36]. The characteristics of the GPS used were a 100 Hz accelerometer, with 16 G Tri-Axial-Track impacts, accelerations, and decelerations; 50 Hz Tri-Axial magnetometers. The GPS size dimensions were (74 mm × 42 mm × 16 mm). The GPS was water-resistant and used infra-red and weighed 56 g for data transmission. 

Before the matches, specifically during the warm-up, the green and red lights were turned on for GPS tracking. GPS devices were placed vertically in the belt bag. After the end of each match, the GPS unit was removed. Then, in the dock station, data was transferred from the GPS device to the computer by the AMS updated software. 

All data was set and collected by default zone in the SPI IQ Absolutes. The following variables were analyzed: duration, MPA, AccZ1 (<2 m·s^−2^); AccZ2 (2 to 4 m·s^−2^); AccZ3 (>4 m·s^−2^); DecZ1 (<−2 m·s^−2^); DecZ2 (−2 to −4 m·s^−2^); DecZ3 (>−4 m·s^−2^) [38]. Accelerometer variables categories were determined based on the time spent at the threshold intensity, which is a valid and reliable way to determine the acceleration of team sports activities [39]. Metabolic power calculation was automatically produced by the GPS, and it was based on the previous two original studies regarding this measure [14,15,16]. The MPA was obtained according to the GPS manufacturer’s manual. It was based on an individual’s running speed, acceleration, and deceleration data, which shows the amount of energy consumed by the player per second (W/kg). A very good inter-reliability (3–5%) was recorded for the MPA.

### 2.4. Statistical Analysis

First, descriptive statistics were used for all sample variables. It was used for the means, standard deviation (SD), and confidence interval (CI) of 95%. Second, the Shapiro-Wilk test was used to analyze normality, and Mauchly’s test was used to analyze sphericity. According to the previous tests, and if normality has been assumed, repeated measures analysis of variance (ANOVA) was used with the Bonferroni post hoc to compare match results (win, draw, defeat), if not, ANOVA Friedman and Mann-Whitney tests were used for the same comparisons. In addition, and to compare data from the first half with the second half, a paired sample t-test was used. Results were significant with *p* ≤ 0.05. All statistical analysis was calculated through version 22.0 of the statistical package for the social sciences (SPSS Inc., Chicago, IL, USA).

Through the determination of the magnitude effects by the difference of two population means, which are then divided by the standard deviation from the data, Cohen’s d effect-size (ES) statistic was calculated. The ES magnitude was defined according to a previous study: <0.2 = trivial, 0.2 to 0.6 = small effect, >0.6 to 1.2 = moderate effect, >1.2 to 2.0 = large effect, and >2.0 = very large [40].

## 3. Results

### 3.1. Characteristics of the Weeks and Matches

Table 1 shows the distribution of tournament divisions for the entire weeks of the season that were analyzed.

In weeks 1–5, 17, 34, and 47, there were friendly matches, while in weeks 12, 22, 25, 27–31, 38, and 45, there were no matches.

### 3.2. Analysis and Comparisons of the Full Game and Halves of the Matches

Descriptive results and comparisons between the win, draw, and defeat results of the variables studied with a confidence interval of 95% are presented in Table 2. 

There were significant differences between draw and defeat matches for AccZ1 during the first half (ES = −0.43 [−1.32, 0.46]). There were significant differences between results of win and draw, plus win and defeat for MPA in the first half (ES = 0.22 [−0.62, 1.10]) and (ES = 0.34 [−0.65, 1.22]), respectively. There were significant differences between draws and defeats for AccZ3 in the second half (ES = 1.37 [0.48, 2.25]). There were significant differences between draws and defeats for MPA in the second half (ES = 0.57 [−0.22, 1.35]).

**Figure 2:**
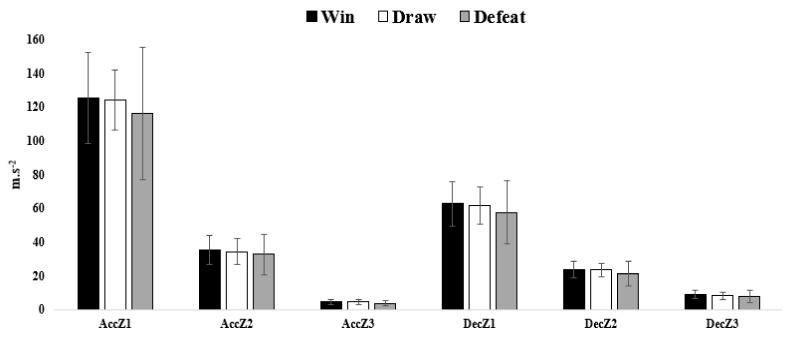
Comparisons based on the match result for accelerations and decelerations. AccZ1, Accelerations in zone 1 (<2 m·s^−2^); AccZ2, accelerations in zone 2 (2 to 4 m·s^−2^); AccZ3, accelerations in zone 3 (>4 m·s^−2^); DecZ1, decelerations in zone 1 (>−2 m·s^−2^); DecZ2, decelerations in zone 2 (−2 to −4 m·s^−2^); DecZ3, decelerations in zone 3 (<−4 m·s^−2^).

Figure 2 also showed the comparisons based on the match results for accelerations and decelerations. No significant differences were found. AccZ1 and AccZ2 were higher in the matches won and lower in the defeated matches. AccZ3 was higher in the drawn matches and lower in defeated matches. DecZ1 and DecZ3 were higher in the matches won and lower in the defeated matches. DecZ2 was higher in the drawn matches and lower in the defeated matches. Although minimal differences were found between the won and drawn matches for the three variables.

### 3.3. First and Second Half Comparisons for Each Result (Win, Draw, and Defeat)

Descriptive results and comparisons between the first and second halves of the variables studied are presented in Table 3. Overall, there were higher values for all variables in the first compared to second half with the exception of AccZ3 in drawn matches. Regarding the matches won, there were significant differences between first and second halves for AccZ2 (ES = 0.89 [0.06, 1.66]), DecZ2 (ES = 0.77 [−0.05, 1.55]), and MPA (ES = 0.66 [−0.15, 1.42]). Regarding the drawn matches, there were significant differences between the first and second halves for duration (ES = 0.63 [−0.18, 1.40]), AccZ1 (ES = 0.75 [−0.07, 1.52]), AccZ2 (ES = 0.60 [−0.20, 1.37]), and DecZ2 (ES = 0.75 [−0.07, 1.52]). Regarding the defeated matches, there were significant differences between the first and second halves for AccZ1 (ES = 1.08 [0.23, 1.87]), AccZ3 (ES = 1.41 [0.51, 2.22]), and MPA (ES = 0.59 [−0.22, 1.35]).

**Figure 3:**
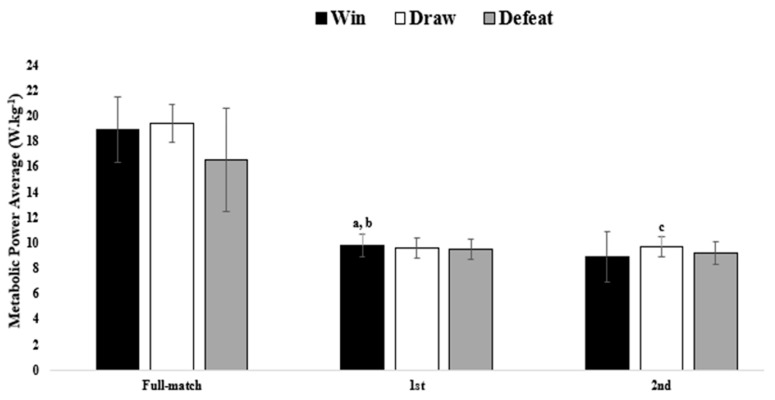
Comparisons based on the match result for metabolic power average between full matches, first and second halves. a denotes difference between win vs draw, b denotes differences between win vs defeat, c denotes differences between draw and defeat, all *p* values considered at levels <0.05.

Descriptive results and comparisons between the wins, draws, and defeats for the full match, first and second halves of the MPA are also presented in Figure 3. The MPA was higher in drawn matches and lower in defeated full matches. The MPA was higher in the matches won than the drawn and defeated first half matches (*p* < 0.05). The MPA was higher in the drawn matches and lower in second-half matches won. There was a significant difference between the drawn and defeated matches (*p* < 0.05). 

## 4. Discussion 

The main aim of this comparative study was to investigate potential links between the external workload parameters of total duration, AccZ1, 2, 3, DecZ1, 2, 3, and MPA with the outcome of the match, and the differences between the first and second halves. The authors hypothesized that the outcome of the match (i.e., win, draw, or defeat) would influence the various workload parameters, and the variables would differ between the two halves of the matches. 

The main findings of the study highlighted differences in the matches drawn in comparison to matches won for the overall duration and the duration of the first half. Draws were overall shorter in full match durations, and within the first half in comparison to wins. This can be impacted by various contextual factors such as the stoppages within the matches and the score-line. The shorter match duration can be indicative of a more even performance by both sides. The even performance can also point to the fact that there was no need for additional time added on within the first half, as often, there is less overall stoppage time added on if there are no significant events happening (e.g., stoppage of play due to an extreme foul, VAR decisions, water break, etc.). 

A winning scoreline can often result in the elongation of the match duration, as often the opposition may be chasing the game, and the winning team may waste time and commit more fouls which may result in more added time. However, this may be the case in matches that are not necessarily dominated by the winning team and are reflective of a more even match battled out until the final minutes. 

Additional findings show that during the first half, there was less AccZ1 in matches that ended in a draw than matches won, and more AccZ3 in the second half for matches drawn than defeated. In addition, the MPA during the first half was less in matches that ended in a draw or a loss in comparison to matches that were won. During the second half, the MPA was higher for matches that ended in a draw in comparison to defeats. The lower overall workload profile observed within the first half of the lesser favorable outcomes (i.e., draws and defeats) points to the potential influence of the lack of intensity from the out-of-possession team, as this lack of intensity may have influenced the lack of various technical/tactical components (e.g., pushing up the pitch, closing space, running in behind, pressing the opponent), that could have resulted in a winning outcome. On the contrary, the second half produced more markers of intensity for the lesser favorable match results of defeats and draws. The likely explanation is the change in the strategy of the team to chase a losing game, and/or making sure the one point obtained is not lost, in the case of a draw, by increasing the physical output in the form of remaining compact to limit space [41]. The findings of this study present some similarities regarding the higher workload outputs in the first half regardless of the match outcome in Iranian professional soccer players [38,42]. 

The within-match analysis revealed greater AccZ2, DecZ2, and MPA in the first vs. second half in matches won. In matches drawn, there were greater duration, AccZ1, and AccZ2, DecZ2 in the first vs. second half. Matches lost, AccZ1 and AccZ3, and MPA were greater in the first vs. the second half. The overall drop in the markers of intensity from the first to second half highlights potential fatigue induced by the match play. The study by Bradley and Noakes [43] highlighted match running performance by way of the total distance covered and high intensity running. This study observed match running performance in 5-min periods, and showed lower performance in the second half compared to the first half [43]. 

The decline in the second half could be a result of a more intense first half, however, a lack of a direct causal relationship between fatigue to a single factor makes it difficult to draw direct conclusions [43,44,45]. Additional factors such as players engaging in pacing strategies to self-regulate throughout the match to avoid fatigue, although there is limited data to explain this theory [43,44,46]. Moreover, the prior knowledge about the first half, opponents and their style of play, and because there is still another half to play may create a higher effort regulation capability, that led players to increase their exercise economy by improving positional relationships, which means that players may have decreased their workload to focus on more ball location, and the space available to play as suggested by Ferraz et al. [47].

A further area of consideration for practitioners is the half-time period. Often, the half-time period constitutes passive activity, where the players try to recover from the first half and engage cognitively with tactical instruction from the coaching staff. This passive period has shown a 2 °C drop in muscle temperature after the half-time break, which resulted in a decline in sprint, jump, and dynamic strength performance [48,49]. This may partially explain the reduced high-intensity efforts in the second half. Additional considerations should be given to the training methodologies utilized by the coaching staff, and how the sessions are designed in relation to the specific match day during the week (i.e., periodization strategies). Although not supported by the scientific literature [43], the success of well-known coaches at the highest level utilizing the concept of tactical periodization [50], has resulted in significant influence on the practical application of this concept. Within a tactical periodization model, each training week looks to incorporate the four dimensions of the game (i.e., technical, tactical, physical, and psychological) in relation to the game model implemented. From a physical standpoint, the “strength days” within a tactical periodization model look to overload the acceleration and deceleration components within the training session using small-sided games of various player densities [51]. The coaches can design training sessions that create a smaller relative pitch area in relation to the number of players in any given space, to allow for short sharp movements to occur in relation to match demands [52]. By further manipulating the work to rest ratios, and the duration of training exercises, coaches can create scenarios where the specific metrics highlighted in this research can be overloaded in relation to the specific phases of play and intensity [51]. This can provide a potential solution to mitigate the drop-off in the specified metrics within the first and second halves, however, further investigation is warranted. 

### Limitations of Study

A large variation can be seen in trends that relate to match-to-match variability for the GPS and WIMU derived variables. Many factors can have an influence on the random nature of the observations within the study. For example, the sample size of the current study was very small (*n* = 10–13), and not many matches were analyzed across the season to establish specific trends (for instance, only three defeats were analyzed). In addition, the formation and the style of play, home vs. away matches, the fitness levels of the players, and training regimen could have an influence on the results [19,53,54,55,56,57]. 

In future studies, the tactical style and the formation of play should be considered in each match in relation to the opposition, standard of opposition, playing position, and situational and environmental factors [19,53,54,55,56,57,58]. In addition, a larger number of matches across multiple seasons to account for a larger sample size can help establish a clearer trend between the aforementioned variables and performance-related outcomes. We also recommend that in future studies, the venue (i.e., home or away) be considered for analysis and review of results. 

## 5. Conclusions

Overall, the first half presented higher values than the second half across the range of values observed, with the exception of MPA. In addition, the matches won showed higher values overall than the matches drawn. The workload profiles observed within the context of the games can help practitioners design more effective training drills via manipulation of space, work to rest ratios, and sets and reps to better prepare players for tactical scenarios in which they need to engage in more physically demanding passages of play. This can take the form of utilizing certain days within the training week (i.e., strength days), to overload the accelerations, decelerations, and related metrics. This can serve as a potential tool to better prepare players for demanding passages of play and combat the drop in intensity observed throughout the match. Future studies should look to implement a league-wide analysis that highlights the observed trends in more than one team to increase the sample size. In addition, similar studies should be replicated across different leagues and levels of competition to highlight any potential differences across various leagues worldwide. 

## Figures and Tables

**Table 1:** Characterization of the weeks and matches included for analysis.

Weeks	Match Result Included
1–5	Not included
6	Draw
7	Win
8	Draw
9	Win
10	Draw
11	Draw
12	Not included
13	Win
14	Win
15	Win
16	Win
17	Not included
18	Win
19	Win
20	Win
21	Draw
22	Not included
23	Draw
24	Win
25	Not included
26	Draw
27–31	Not included
32	Draw
33	Win
34	Not included
35	Defeat
36	Win
37	Draw
38	Not included
39	Draw
40	Win + Draw
41	Defeat
42	Win
43	Draw
44	Win
45	Not included
46	Draw + Win
47	Not included
48	Defeat

**Table 2:** Comparison of full match-day, first and second half data between wins, draws and defeats per squad average, mean ± standard deviation (confidence interval, CI, 95%).

**Full match**	**Draw (CI, 95%)**	**Defeat (CI, 95%)**	**Win (CI, 95%)**
Duration (min), *n* = 13	88.8 ± 11.9 * (81.6–95.9)	84.2 ± 13.7 (75.9–92.5)	82.1 ± 27.4 (65.5–98.6)
AccZ1 (m·s^−2^), *n* = 13	124.2 ± 17.8 (113.5–135.0)	116.1 ± 39.3 (92.3–139.8)	125.4 ± 27.1 (109.0–141.7)
AccZ2 (m·s^−2^), *n* = 13	34.2 ± 7.6 (29.6–38.8)	32.7 ± 12.1 (25.4–40.0)	35.4 ± 8.4 (30.3–40.5)
AccZ3 (m·s^−2^), *n* = 12	4.6 ± 1.6 (3.6–5.6)	3.7 ± 1.7 (2.7–4.89	4.4 ± 1.5 (3.4–5.4)
DecZ1 (m·s^−2^), *n* = 13	61.6 ± 10.9 (55.0–68.2)	57.5 ± 18.7 (46.2–69.0)	62.8 ± 13.2 (54.8–70.8)
DecZ2 (m·s^−2^), *n* = 13	23.6 ± 3.9 (21.3–26.0)	21.2 ± 7.1 (16.9–25.5)	23.4 ± 4.9 (20.5–26.4)
DecZ3 (m·s^−2^), *n* = 13	8.2 ± 2.2 (6.9–9.5)	7.7 ± 3.6 (5.5–9.8)	9.0 ± 2.2 (7.7–10.3)
MPA (W·kg^−1^), *n* = 13	19.4 ± 1.5 (18.5–20.3)	16.5 ± 4.1 (14.0–18.9)	18.9 ± 2.6 (17.3–20.5)
**First half**	**Draw (CI, 95%)**	**Defeat (CI, 95%)**	**Win (CI, 95%)**
Duration (min), *n* = 13	47.2 ± 0.2 ** (46.7–47.7)	46.3 ± 1.2 (43.5–49.1)	48.6 ± 0.2 (48.1–49.0)
AccZ1 (m·s^−2^), *n* = 10	67.8 ± 10.4 * (60.3–75.2)	73.0 ± 12.7 (63.9–82.1)	72.5 ± 15.5 (61.4–83.6)
AccZ2 (m·s^−2^), *n* = 10	18.2 ± 5.2 (14.5–22.0)	19.8 ± 5.0 (16.2–23.4)	19.8 ± 5.4 (15.9–23.7)
AccZ3 (m·s^−2^), *n* = 10	2.2 ± 0.9 (1.6–2.8)	2.8 ± 1.1 (2.0–3.5)	2.5 ± 1.1 (1.7–3.2)
DecZ1 (m·s^−2^), *n* = 10	33.0 ± 7.2 (27.9–38.1)	34.4 ± 7.9 (28.8–40.0)	34.2 ± 8.5 (28.2–40.2)
DecZ2 (m·s^−2^), *n* = 10	12.7 ± 3.4 (10.3–15.1)	12.7 ± 2.0 (11.2–14.2)	12.8 ± 3.6 (10.2–15.3)
DecZ3 (m·s^−2^), *n* = 10	4.1 ± 0.9 (3.5–4.8)	4.6 ± 1.7 (3.3–5.8)	4.4 ± 1.2 (3.6–5.3)
MPA (W·kg^−1^), *n* = 10	9.6 ± 0.8 ** (9.0–10.1)	9.5 ± 0.8 *** (8.9–10.1)	9.8 ± 0.9 (9.2–10.5)
**Second half**	**Draw (CI, 95%)**	**Defeat (CI, 95%)**	**Win (CI, 95%)**
Duration (min), *n* = 13	43.7 ± 7.6 (39.0–48.3)	39.8 ± 8.0 (35.0–44.7)	44.7 ± 10.1 (28.6–50.9)
AccZ1 (m·s^−2^), *n* = 13	58.3 ± 8.7 (53.0–63.5)	59.9 ± 12.5 (52.4–67.5)	56.9 ± 14.2 (48.4–65.5)
AccZ2 (m·s^−2^), *n* = 13	15.8 ± 3.0 (14.0–17.6)	17.5 ± 5.4 (14.2–20.8)	15.6 ± 4.1 (13.2–18.1)
AccZ3 (m·s^−2^), *n* = 12	2.4 ± 0.8 * (1.9–3.0)	1.4 ± 0.6 (1.0–1.8)	2.0 ± 0.6 (1.6–2.4)
DecZ1 (m·s^−2^), *n* = 13	29.3 ± 4.3 (26.7–31.9)	31.1 ± 5.6 (27.7–34.5)	29.2 ± 6.2 (25.5–32.9)
DecZ2 (m·s^−2^), *n* = 13	10.9 ± 1.6 (9.9–11.9)	11.4 ± 3.2 (9.5–13.4)	10.6 ± 2.6 (9.0–12.1)
DecZ3 (m·s^−2^), *n* = 13	3.8 ± 0.8 (3.3–4.3)	4.2 ± 1.9 (3.0–5.3)	4.2 ± 1.1 (3.6–4.8)
MPA (W·kg^−1^), *n* = 13	9.7 ± 0.8 * (9.3–10.2)	9.2 ± 0.9 (8.6–9.7)	8.9 ± 2.0 (7.7–10.2)

AccZ1, Accelerations in zone 1 (<2 m·s^−2^); AccZ2, accelerations in zone 2 (2 to 4 m·s^−2^); AccZ3, accelerations in zone 3 (>4 m·s^−2^); DecZ1, decelerations in zone 1 (>−2 m·s^−2^); DecZ2, decelerations in zone 2 (−2 to −4 m·s^−2^); DecZ3, decelerations in zone 3 (<−4 m·s^−2^); MPA, metabolic power average; m∙s^−^^2^, meter per second squared; W∙kg^−^^1^, Watts per kilogram; CI 95%, confidence interval level of 95 percentage; * significant differences between draw vs defeat, *p* < 0.05; ** significant differences between draw vs win, *p* < 0.05; *** significant differences between defeat vs win, *p* < 0.05.

**Table 3:** Comparison of first and second halves data for the matches won, drawn, and defeated per squad average, mean ± standard deviation (confidence interval, CI, 95%).

**Matches result (Win)**	**First half (CI, 95%)**	**Second half (CI, 95%)**	***p***
Duration (min), *n* = 10	45.1 ± 7.9 (40.3–49.9)	43.7 ± 7.6 (39.0–48.3)	0.613
AccZ1 (m·s^−2^), *n* = 13	68.4 ± 20.9 (55.8–81.1)	56.9 ± 14.2 (48.4–65.5)	0.100
AccZ2 (m·s^−2^), *n* = 13	19.8 ± 5.3 (16.6–23.0)	15.6 ± 4.1 (13.2–18.1)	0.005 *
AccZ3 (m·s^−2^), *n* = 13	2.4 ± 1.0 (1.8–3.0)	2.0 ± 0.6 (1.6–2.4)	0.080
DecZ1 (m·s^−2^), *n* = 13	33.6 ± 9.7 (27.7–39.4)	29.2 ± 6.2 (25.5–32.9)	0.118
DecZ2 (m·s^−2^), *n* = 13	12.9 ± 3.3 (10.9–14.9)	10.6 ± 2.6 (9.0–12.1)	0.029 *
DecZ3 (m·s^−2^), *n* = 13	4.8 ± 1.5 (3.9–5.7)	4.2 ± 1.1 (3.6–4.8)	0.180
MPA (W·kg^−1^), *n* = 13	9.9 ± 0.8 (9.4–10.4)	8.9 ± 2.0 (7.7–10.2)	0.048 *
**Matches result (Draw)**	**First half (CI, 95%)**	**Second half (CI, 95%)**	***p***
Duration (min), *n* = 10	44.4 ±6.5 (40.4–48.3)	39.8 ± 8.0 (35.0–44.7)	0.008 *
AccZ1 (m·s^−2^), *n* = 13	65.9 ± 11.5 (59.0–72.0)	58.3 ±8.7 (53.0–63.5)	0.017 *
AccZ2 (m·s^−2^), *n* = 13	18.4 ±5.3 (15.2–21.5)	15.8 ±3.0 (14.0–17.6)	0.040 *
AccZ3 (m·s^−2^), *n* = 13	2.1 ± 0.8 (1.6–2.6)	2.4 ± 0.8 (1.9–2.9)	0.140
DecZ1 (m·s^−2^), *n* = 13	32.3 ± 7.5 (27.8–36.8)	29.3 ± 4.3 (26.7–31.9)	0.077
DecZ2 (m·s^−2^), *n* = 13	12.7 ± 3.0 (10.9–14.5)	10.9 ± 1.6 (9.9–11.9)	0.037 *
DecZ3 (m·s^−2^), *n* = 13	4.4 ± 1.5 (3.5–5.3)	3.8 ± 0.8 (3.3–4.3)	0.079
MPA (W·kg^−1^), *n* = 13	9.6 ± 0.7 (9.2–10.1)	9.7 ± 0.8 (9.3–10.2)	0.312
**Matches result (Defeat)**	**First half (CI, 95%)**	**Second half (CI, 95%)**	***p***
Duration (min), *n* = 10	48.6 ± 0.6 (48.1–49.0)	47.1 ± 7.4 (41.8–52.4)	0.537
AccZ1 (m·s^−2^), *n* = 10	73.0 ± 12.7 (63.9–82.1)	60.7 ± 9.9 (53.6–67.8)	0.001 *
AccZ2 (m·s^−2^), *n* = 10	19.8 ± 5.0 (16.2–23.4)	18.1 ± 4.8 (14.6–21.5)	0.413
AccZ3 (m·s^−2^), *n* = 10	2.8 ± 1.1 (2.0–3.5)	1.5 ± 0.7 (1.0–2.0)	0.018 *
DecZ1 (m·s^−2^), *n* = 10	34.4 ± 7.9 (28.7–40.0)	31.3 ± 5.2 (27.6–35.0)	0.181
DecZ2 (m·s^−2^), *n* = 10	12.7 ± 2.0 (11.2–14.2)	11.8 ± 2.4 (10.0–13.5)	0.393
DecZ3 (m·s^−2^), *n* = 10	4.6 ± 1.7 (3.3–5.8)	4.4 ± 1.8 (3.1–5.7)	0.824
MPA (W·kg^−1^), *n* = 10	9.5 ± 0.8 (8.9–10.1)	9.0 ± 0.9 (8.4–9.6)	0.003 *

AccZ1, AccZ1, Accelerations in zone 1 (<2 m·s^−2^); AccZ2, accelerations in zone 2 (2 to 4 m·s^−2^); AccZ3, accelerations in zone 3 (>4 m·s^−2^); DecZ1, decelerations in zone 1 (>−2 m·s^−2^); DecZ2, decelerations in zone 2 (−2 to −4 m·s^−2^); DecZ3, decelerations in zone 3 (<−4 m·s^−2^); MPA, metabolic power average; m∙s^−^^2^, meter per second squared; W∙kg^−^^1^, Watts per kilogram; CI 95%, confidence interval level of 95 percentage; * denotes difference from the second half. All *p* values considered at levels < 0.05.

## Data Availability

The datasets used and/or analyzed during the current study are available from the corresponding author on reasonable request.
